# Advancing Pyrogen Testing for Vaccines with Inherent Pyrogenicity: Development of a Novel Reporter Cell-Based Monocyte Activation Test (MAT)

**DOI:** 10.3390/vaccines13101009

**Published:** 2025-09-26

**Authors:** Sijia Yi, Jenny Xu, Liping Song, Frank Celeste, Christopher J. Wang, Melissa C. Whiteman

**Affiliations:** 1 Analytical Research and Development, Merck & Co., Inc., West Point, PA 19486, USA; jingyuan_xu@merck.com (J.X.); frank.celeste@merck.com (F.C.); christopher_wang@merck.com (C.J.W.); melissa.whiteman@merck.com (M.C.W.); 2 Biostatistics and Research Decision Sciences, Merck & Co., Inc., West Point, PA 19486, USA; liping_song@merck.com

**Keywords:** pyrogenicity, monocyte activation test (MAT), reporter cell line, outer membrane protein complex (OMPC), vaccine safety, 3Rs

## Abstract

Background/Objectives: Pyrogens, fever-inducing substances from biological or environmental sources, are recognized by Toll-like receptors (TLRs) predominantly expressed by human monocytes and represent a critical quality attribute (CQA) for pharmaceutical safety. The rabbit pyrogen test (RPT), widely used for pyrogen assessment, suffers from high variability, limited accuracy, and poor reproducibility, particularly for vaccines containing inherent pyrogens such as outer membrane protein complex (OMPC)-based vaccines. Existing in vitro alternatives using peripheral blood mononuclear cells (PBMCs) are challenged by donor-to-donor variability and the operational complexity of ELISA readouts. To support the 3Rs (Refinement, Reduction, Replacement) and provide a more reliable quality control (QC) method, we developed a reporter cell–based monocyte activation test (MAT) suitable for release testing. Methods: We screened human monocytic reporter cell lines engineered with NFκB-responsive promoter elements driving a luminescent reporter. Reporter cells were treated with diverse endotoxin and non-endotoxin pyrogens and luminescence was quantified after stimulation. Selected THP-1-derived reporter cells were used to develop an MAT for OMPC. Assay performance was evaluated following validation guidelines: linearity, accuracy, precision, analytical range (relative to a reference lot), and robustness under deliberate parameter variations. Results: The THP-1 reporter cells could detect a wide range of pyrogens via simple luminescence readouts. For OMPC testing, the MAT demonstrated strong linearity (R^2^ ≥ 0.99), accuracy with relative bias within ±10.3%, and high precision (overall %RSD ≤ 6.9%) across the 25–300% range. Deliberate variations in assay parameters did not materially affect performance, indicating robustness appropriate for routine release testing. Conclusions: The implementation of reporter cell-based MAT assays enhances consistency, reliability, and efficiency in evaluating the pyrogenicity and safety of drug products, supporting global initiatives to minimize animal testing while ensuring regulatory compliance.

## 1. Introduction

Pyrogens can originate from various microorganisms and environmental contaminants, including components from Gram-negative and Gram-positive bacteria, viruses, fungi, yeast, and environmental particles [1]. Pyrogen contamination in pharmaceutical products can lead to severe reactions, such as multiple organ failure, shock, and even death in extreme cases [2]. To mitigate the risk of febrile reactions following administration, it is critical to control the amount of pyrogenic components in parenteral medicines, such as vaccines. Given that components from microorganisms are potent immune stimulators and are widely used in vaccine development, it is essential to regulate the amount of pyrogenic components used in humans. Regulatory requirements mandate that pyrogens be tested for all pharmaceutical products and devices prior to release [3] (21 CFR 211.167(a); 21 CFR 610.13(b)).

The rabbit pyrogen test (RPT), introduced in the early 20th century, has been widely used to evaluate and detect various types of pyrogen contaminants [4]. As a qualitative assay, the RPT was initially employed to detect the presence of contaminating pyrogens in parenteral preparations by monitoring temperature changes in rabbits following intravenous administration of a test sample [4]. Since the 1980s, the RPT has been used as a pyrogen release assay to determine the pyrogenicity of vaccines [5,6]. However, this approach to RPT for vaccines with inherent pyrogens, such as meningococcal membrane protein-based vaccines, is problematic due to the route of administration (intravenous for RPT versus intramuscular for humans) and the necessary additional dilution of the human dose until a pyrogenic response is no longer detected in rabbits [4]. Furthermore, the RPT demonstrates low reproducibility or accuracy, lacks harmonization across, strains, and ages of rabbits in different regions, and raises ethical concerns regarding animal use [4,7,8]. The bacterial endotoxin test or Limulus amebocyte lysate (LAL) test [9,10] is an in vitro pyrogen test specific for endotoxins but is not suitable for other non-endotoxin pyrogens and may be affected by sample components [11].

To overcome these challenges and align with global commitments to reduce animal usage based on the 3Rs principle (Refinement, Reduction, Replacement), the in vitro MAT has been incorporated into the European Pharmacopoeia (Chapter 2.6.30 and 2.6.40) [12,13] and United States Pharmacopeia (USP General Chapter <151>) [10] as an alternative to the RPT for evaluating pyrogenicity [14]. Human immune cells, such as whole blood or peripheral blood mononuclear cells (PBMCs), have become the most commonly used cell sources in the MAT assay [15,16,17]. When exposed to PBMCs, pyrogens are recognized by pattern recognition receptors (PRRs), such as Toll-like receptors (TLRs), on heterogeneous immune cells, resulting in the activation of nuclear factor-kappa B (NFκB) and subsequent production and release of inflammatory cytokines, chemokines, and other molecules involved in the immune response. In particular, the febrile reaction is mediated by interleukin-6 (IL-6), interleukin-1β (IL-1β), and tumor necrosis factor α (TNFα), whose expression is dependent on NFκB engagement [18]. The released inflammatory cytokines, such as IL-1β and IL-6, can be further quantified using ELISA assays. However, the inherent variability in PBMCs from different donors and the multiple steps involved in the ELISA assay pose challenges for method validation and quality control (QC) testing [19]. Additionally, various human monocytic cell lines (e.g., THP-1, MonoMac 6 (MM6)) have been explored and demonstrated their suitability for detecting pyrogens in the MAT [20,21].

To address these challenges and develop a reliable and QC-friendly MAT assay, we have screened and identified reporter cells with NFκB-responsive promoter elements driving the expression of reporter genes in various human monocytic cells for pyrogen testing. The expression of TLR receptors has been evaluated using RNA sequencing and flow cytometry analysis, and their responses to diverse pyrogens (both endotoxins and non-endotoxin pyrogens) were assessed through cell-based assays. We demonstrated that reporter cells derived from THP-1, which outperform other monocyte-derived (U937) reporter cells, can quantitatively, accurately, and consistently detect a wide range of pyrogens by simply measuring luminescence after pyrogen stimulation. We developed a pyrogen release assay using the reporter cell-based MAT specifically for vaccines with inherent pyrogens, such as OMPC-based vaccines. In addition to the traditional propagated cell model, the ready to use (RTU) cell model [22], which is used directly from frozen storage, is also explored and implemented in MAT to reduce variability, simplify workflows, and facilitate laboratory transfers. The implementation of reporter cell-based MAT assays provides the pharmaceutical industry with improved consistency, reliability, and efficiency in evaluating the pyrogenicity and safety of drug products.

## 2. Materials and Methods

### 2.1. Materials

All consumables and reagents used for cell culture or cell-based assays were purchased as sterile and pyrogen-free. Unless otherwise stated, consumables and reagents for cell culture were obtained from Thermo Fisher Scientific (Waltham, MA, USA). All anti-human monoclonal antibodies and reagents for flow cytometry analysis were sourced from BioLegend (San Diego, CA, USA), unless otherwise indicated. The TLR agonists, including Pam3CSK4 (TLR1/TLR2), LTA-SA (TLR2), HKLM (TLR2), Poly(I:C) HMW and Poly(I:C) LMW (TLR3), FLA-ST ultrapure (TLR5), FSL-1 (TLR2/TLR6), R848 (Resiquimod) (TLR7/8), Imiquimod (R837) (TLR7), and synthetic CpG oligodeoxynucleotide ODN 2006 (ODN 7909) (TLR9), were purchased from InvivoGen (San Diego, CA, USA). The United States Pharmacopeia (USP) reference standard endotoxin was obtained from either Lonza (Basel, Switzerland) or Sigma Aldrich (St. Louis, MO, USA). All TLR agonists were reconstituted in sterile, endotoxin-free water and stored according to the manufacturer’s instructions.

OMPC materials and the formulation buffer were provided by Merck & Co., Inc., West Point, PA, USA manufacturing site.

### 2.2. Methods

#### Cell Culture

U937-NFkB-NLuc cells were generated in-house (Merck & Co., Inc., Rahway, NJ, USA) and maintained at 37 °C in a humidified atmosphere containing 5% CO_2_. The cells were cultured in RPMI 1640 medium supplemented with GlutaMAX, HEPES (Thermo Fisher Scientific, Waltham, MA, USA), 10% heat-inactivated fetal bovine serum (Sigma Aldrich, St. Louis, MO, USA), 100 U/mL of Penicillin/Streptomycin, and 600 µg/mL of Hygromycin (Thermo Fisher Scientific, Waltham, MA, USA). The cells were maintained at a density of 0.8 × 10^5^ to 2 × 10^6^ cells/mL and passaged every 2 to 3 days.

THP1-Lucia NFκB cells (InvivoGen, San Diego, CA, USA) were derived from the human THP-1 monocyte cell line through stable integration of an NF-κB inducible Lucia reporter construct. The cells were cultured according to the manufacturer’s instructions. Briefly, they were maintained at 37 °C in a humidified atmosphere with 5% CO_2_ in RPMI 1640 medium supplemented with 2 mM L-glutamine, 25 mM HEPES, 10% (*v*/*v*) heat-inactivated fetal bovine serum, 100 U/mL penicillin, 100 µg/mL streptomycin, and 100 µg/mL Normocin (Thermo Fisher Scientific, Waltham, MA, USA). The cells were maintained at a density of 7 × 10^5^ to 2 × 10^6^ cells/mL and passaged every 3 to 4 days.

TLR Bioassay cells (Promega, Madison, WI, USA) were derived from human monocytic cells that endogenously express TLRs and contain a stably integrated NanoLuc (NL) luciferase reporter driven by TLR pathway-dependent response elements. The cells were cultured according to the manufacturer’s instructions. Briefly, they were maintained at 37 °C in a humidified atmosphere with 5% CO_2_ in RPMI 1640 medium supplemented with L-glutamine and HEPES, 10% (*v*/*v*) heat-inactivated fetal bovine serum, and 200 µg/mL hygromycin B (Thermo Fisher Scientific, Waltham, MA, USA). The cells were passaged every 2 to 3 days and maintained at a density of 5 × 10^5^ to 1 × 10^6^ cells/mL.

### 2.3. RNAseq

#### 2.3.1. Library Preparation and Sequencing

PBMCs (ATCC, Manassas, VA, USA), U937-NFkB-NLuc cells, THP1-Lucia NFκB cells, TLR Bioassay cells, and Mono-Mac-6 (MM6) cells (Merck Millipore, Burlington, MA, USA) were lysed and total RNA was extracted using Qiagen Rneasy Plus Mini kit (Qiagen, Hilden, Germany) per manufacturer’s instructions. RNA samples were quantified using Qubit 2.0 Fluorometer (Life Technologies, Carlsbad, CA, USA) and ribonucleic acid (RNA) integrity was checked using Agilent TapeStation 4200 (Agilent Technologies, Palo Alto, CA, USA). ERCC RNA Spike-In Mix (Thermo Fisher Scientific, Waltham, MA, USA) was added to normalized total RNA prior to library preparation following manufacturer’s protocol.

RNA sequencing libraries were prepared using the NEBNext Ultra II RNA Library Prep Kit (New England Biolabs, Ipswich, MA, USA) under manufacturer’s instructions. Briefly, messenger RNAs (mRNAs) were initially enriched with Oligod(T) beads. Enriched mRNAs were fragmented for 15 min at 94 °C. First strand and second strand complementary deoxyribonucleic acid (cDNA) were subsequently synthesized using primers which included random priming sites and Unique Molecular Identifiers (UMIs). UMI sequences were incorporated in the resultant cDNA. cDNA fragments were end-repaired and adenylated at 3′ ends. Universal adapters were ligated to cDNA fragments, followed by index addition and library enrichment by polymerase chain reaction (PCR) with limited cycles. The sequencing library was validated on the Agilent TapeStation (Agilent Technologies, Palo Alto, CA, USA) and quantified by using Qubit 2.0 Fluorometer (Invitrogen, Carlsbad, CA) well as by quantitative PCR (KAPA Biosystems, Wilmington, MA, USA).

Sequencing libraries were clustered on a flowcell. After clustering, the flowcell was loaded on the Illumina NovaSeq instrument (Illumina, San Diego, CA, USA) according to manufacturer’s instructions. The samples were sequenced using a 2 × 150 bp Paired End (PE) configuration. Image analysis and base calling were conducted with the Control software by Azenta (South Plainfield, NJ, USA). Raw sequence data (.bcl files) generated by the sequencer were converted into fastq files and de-multiplexed.

#### 2.3.2. Data Processing and Analysis

Sequence reads were trimmed to remove adapter sequences and low-quality bases using fastp (v.0.23.1). UMI-based de-duplication was performed using fastp (v.0.23.1). The trimmed and de-duplicated reads were then mapped to the Homo sapiens GRCh38 with ERCC genes reference genome available on ENSEMBL using the STAR aligner v.2.5.2b. BAM files were generated as a result of this step. Unique gene hit counts were calculated by using feature Counts from the Subread package v.1.5.2. Only unique reads that fell within exon regions were counted. Counts for each gene were divided by the gene length (in kilobases) to obtain reads per kilobase (RPK). Each gene’s RPK was then divided by the sum of RPKs in the sample and multiplied by 10^6^ to yield transcripts per million (TPM).

### 2.4. Flow Cytometric Analysis of TLR Expression

Frozen peripheral blood mononuclear cells (PBMCs) (ATCC, Manassas, VA, USA), THP1-Lucia-NFκB cells (InvivoGen, San Diego, CA, USA), and TLR Bioassay cells (Promega, Madison, WI, USA) were washed with Phosphate-Buffered Saline (PBS) and plated in 96-well V-bottom plates at a density of 1 × 10^6^ cells per well. The cells were initially stained with Human TruStain FcX™ and Zombie Aqua live/dead dye for 30 min at 4 °C in the dark. Subsequently, the cells were stained for an additional 30 min at 4 °C in the dark with either isotype control or specific monoclonal antibodies, including PE Mouse IgG2a, κ Isotype Control (FC) antibody, PE anti-human CD282 (TLR2) antibody, PE anti-human CD284 (TLR4) antibody, PE anti-human CD285 (TLR5) antibody, PE anti-human CD286 (TLR6) antibody, and PE anti-human CD281 (TLR1) (GD2.F4), as well as PE Mouse IgG1 kappa Isotype Control (P3.6.2.8.1) from Thermo Fisher Scientific (Waltham, MA, USA). The stained cells were washed twice with 200 µL of staining buffer per well, resuspended in staining buffer, and fixed with fixation buffer. The cells were then analyzed using a Cytoflex flow cytometer (Beckman Coulter, Indianapolis, IN, USA). Data were acquired from 20,000 cells per sample and analyzed using FlowJo v10.9.0.

### 2.5. Cell-Based Assay

#### U937-NFkB-NLuc Cells

U937-NFkB-NLuc cells were plated in 96-well white culture plates (Revvity, Waltham, MA, USA) at an optimized density of 9.0 × 10^4^ cells per well in 50 µL of cell suspension, which was thoroughly mixed prior to plating. The TLR agonists were pre-diluted as follows: USP RSE to 2000 EU/mL, Pam3CSK4 to 5 µg/mL, LTA-SA to 50 µg/mL, FSL-1 to 1 µg/mL, Poly(I:C) HMW to 20 µg/mL, Poly(I:C) LMW to 20 µg/mL, ultrapure FLA-ST to 0.2 µg/mL, Imiquimod to 20 µg/mL, ssRNA40 to 20 µg/mL, and ODN2006 to 20 µg/mL. Each stock was then serially diluted 3-fold in assay medium to generate 8 concentration points for each dose–response curve in 96-well dilution plates. Subsequently, 50 µL of the diluted samples were transferred from the dilution plate to the 96-well white assay plate containing the cells. Following a 5 h incubation at 37 °C, the Nano-Glo Luciferase Assay Reagent (Promega, Madison, WI, USA) was prepared by thawing the substrate and mixing it with the buffer in a 1:50 ratio. After the initial incubation, the plates were allowed to equilibrate to room temperature for 10 min before adding 100 µL of the reconstituted reagent to each well. The plates were then covered with foil, shaken for 10 min at room temperature, and luminescence was measured using a SpectraMax i3x microplate reader (Molecular Devices, San Jose, CA, USA).

### 2.6. THP1-Lucia NFκB Cells

THP1-Lucia NFκB cells were plated in Costar 96-well cell culture plates (Corning, New York, NY, USA) at a density of 1.0 × 10^5^ cells per well in 180 µL of thoroughly mixed cell suspension. The plates were incubated at 37 °C for 5 h. The TLR agonists were pre-diluted as follows: USP RSE to 400 EU/mL, Pam3CSK4 to 2 µg/mL, HKLM to 5.0 × 10^8^ cells/mL, LTA-SA to 20 µg/mL, FSL-1 to 1 µg/mL, Poly(I:C) HMW to 50 µg/mL, Poly(I:C) LMW to 50 µg/mL, ultrapure FLA-ST to 5 µg/mL, R848 to 50 µg/mL, and ODN2006 to 20 µg/mL. Each stock was then serially diluted 3-fold in assay medium to generate 12 concentration points for each dose–response curve in 96-well dilution plates. 50 µL of each serially diluted samples were transferred into the corresponding wells of 96-well white assay plates, which were incubated for approximately 22 ± 2 h at 37 °C. A QUANTI-Luc 4 Reagent (InvivoGen, San Diego, CA, USA) working solution was prepared by diluting 1.25 mL of concentrated reagent in 23.75 mL of Hypure cell culture-grade water, followed by brief vortexing. After incubation, 20 µL of cell culture medium from each well was transferred to a 96-well white culture plate (Revvity, Waltham, MA, USA), followed by the addition of 50 µL of QUANTI-Luc 4 Reagent (1X) to each well. The plate was gently tapped to mix and read immediately using the SpectraMax i3x microplate reader (Molecular Devices, San Jose, CA, USA).

### 2.7. TLR Bioassay Cells

TLR Bioassay cells were plated in 96-well white culture plates (Revvity, Waltham, MA, USA) at a density of 5.0 × 10^4^ cells per well for the propagation model or 3.0 × 10^4^ cells per well for the RTU model, in 60 µL of thoroughly mixed cell suspension. The plates were covered and incubated overnight at 37 °C with 5% CO_2_. Following incubation, assay medium (10% FBS in RPMI-1640) was warmed to 37 °C. The TLR agonists were pre-diluted as follows: USP RSE to 400 EU/mL, Pam3CSK4 to 800 ng/mL, HKLM to 2 × 10^9^ cells/mL, LTA-SA to 20 µg/mL, FSL-1 to 400 ng/mL, Poly(I:C) HMW to 100 µg/mL, Poly(I:C) LMW to 100 µg/mL ultrapure FLA-ST to 2 µg/mL, R848 to 0.2 mg/mL, and ODN2006 to 50 µg/mL. Each stock was then serially diluted 3-fold in assay medium to generate 12 concentration points for each dose–response curve in 96-well dilution plates. 20 µL of each serially diluted sample were transferred into the corresponding wells of the assay plates, which were incubated for approximately 4 h at 37 °C. The Bio-Glo-NL Luciferase assay reagent (Promega, Madison, WI, USA) was prepared by thawing the buffer and mixing 0.4 mL of substrate with 20 mL of buffer. After removing the assay plates from the incubator, they were allowed to equilibrate to room temperature for 15 min. Subsequently, 80 µL of Bio-Glo-NL Reagent was added to each well. The plates were shaken for 10 min prior to measuring luminescence in Cytation 7 multimode reader (Agilent Technologies, Winooski, VT, USA).

### 2.8. Peripheral Blood Mononuclear Cells (PBMCs)

Cryopreserved pooled PBMCs were isolated and purified from four healthy donors with monocytes more than 10% (ATCC, Manassas, VA, USA). The TLR agonists were pre-diluted as follows: USP RSE to 10 EU/mL, Pam3CSK4 to 1 µg/mL, HKLM to 5 × 10^8^ cells/mL, LTA-SA to 20 µg/mL, FSL-1 to 50 ng/mL, Poly(I:C) HMW to 100 µg/mL, Poly(I:C) LMW to 100 µg/mL, ultrapure FLA-ST to 50 ng/mL, Imiquimod to 50 µg/mL, R848 to 50 µg/mL, and ODN2006 to 50 µg/mL. Each stock was then serially diluted 3-fold in assay medium to generate 11 concentration points for each dose–response curve in 96-well dilution plates. In a 96-well assay plate, 100 µL of serially diluted positive controls and samples were added to the corresponding wells, and the plate was incubated at 37 °C while preparing PBMCs. Vials of PBMCs were thawed in a 37 °C water bath, transferred to a conical centrifuge tube containing assay medium, and centrifuged at 340× *g* for 10 min. The cell pellet was resuspended in 10 mL of assay medium, mixed, and counted using a hemocytometer [23]. Subsequently, 100 µL containing 1.0 × 10^6^ cells were added to each well of the assay plate. The plates were incubated for 22 ± 2 h before preparing the AlphaLISA IL-6 kit solutions (Revvity, Waltham, MA, USA). Following incubation, 40 µL of a 2.5× AlphaLISA Anti-IL-6 Acceptor Bead solution was added to each well, followed by 10 µL of supernatant from the tissue culture plates, ensuring careful removal of supernatant to avoid disturbing the cells. The mixture was incubated at ambient temperature in a biosafety cabinet for 60 ± 10 min. In a dark environment, a 2× Streptavidin donor bead solution was prepared and added to each well, followed by a 30 ± 5 min incubation. Finally, the plate was read using an EnVision plate reader (Revvity, Waltham, MA, USA) for AlphaLISA analysis.

### 2.9. Product-Specific MAT Method Development for OMPC

The MAT assay was developed for the OMPC in accordance with the guidelines of the European Pharmacopoeia (Ph. Eur. 2.6.30 and Ph. Eur. 2.6.40) and ICH Q2 (R1). TLR Bioassay cells were used as the MAT test system, and the assay procedure was optimized using the specific product—OMPC. Released commercial batches of OMPC materials that were safe, having passed the Rabbit Pyrogen Test (RPT), and were representative of the product were used for the study. The TLR Bioassay RTU cell vials were removed from liquid nitrogen storage and immediately thawed at 37 °C for 2–3 min. Cells were plated into white 96-well culture plates (Revvity, Waltham, MA, USA) at 3.0 × 10^4^ cells per well in 60 µL of thoroughly mixed cell suspension. Plates were covered and incubated overnight at 37 °C with 5% CO_2_. After incubation, assay medium (RPMI-1640 with 10% FBS) was warmed to 37 °C. OMPC was pre-diluted to 226.8 µg/mL of protein concentration, and USP RSE was pre-diluted to 400 EU/mL. Both OMPC and USP RSE were then serially diluted 2.2-fold in assay medium to yield 11 concentration points per dose–response curve in 96-well dilution plates. 20 µL of each diluted sample were transferred to the corresponding assay wells, which were incubated at 37 °C for 4 h. The Bio-Glo-NL Luciferase assay reagent (Promega, Madison, WI, USA) was prepared by thawing the buffer and mixing 0.4 mL of substrate with 20 mL of buffer. After removing the assay plates from the incubator, they were allowed to equilibrate to room temperature for 15 min. Subsequently, 80 µL of Bio-Glo-NL Reagent was added to each well. The plates were shaken for 10 min prior to measuring luminescence in Cytation 7 multimode reader (Agilent Technologies, Winooski, VT, USA).

The assay performance parameters: linearity, precision (repeatability and intermediate precision), accuracy, range, and specificity were assessed based on the relative pyrogenicity of the test samples. Multiple solutions were prepared through serial dilution from a reference batch to establish a reference curve. Mock OMPC samples representing five different target levels (300%, 200%, 100%, 50%, and 25%) were generated from this reference. Each sample was tested in at least two independent runs, with two to four plates per run, conducted by two analysts on different days.

The matrix buffers with or without OMPC, were tested in the MAT to determine specificity. To evaluate the detection of both endotoxins and non-endotoxin pyrogenic contaminants in OMPC, TLR Bioassay cells were stimulated with serially diluted USP reference standard endotoxin (RSE) and LTA-SA at defined amounts, separately. In the same experiment, the TLR Bioassay cells were stimulated with serially diluted OMPC, with or without the addition of defined amounts of pyrogens (USP RSE, LTA-SA). Specifically, OMPC was pre-diluted in assay medium at a 1:50 ratio. USP RSE was pre-diluted to 2000 EU/mL, 1000 EU/mL, and 400 EU/mL. LTA-SA was pre-diluted to 20 µg/mL, 10 µg/mL, and 5 µg/mL. Aliquots of each USP RSE or LTA-SA concentration (or assay medium as the control) were then spiked into the pre-diluted OMPC at a 1:1 ratio. These mixtures were transferred to 96-well dilution plates and serially diluted 2-fold in assay medium to generate 12 concentration points per dose response curve. The same procedure was subsequently followed for the MAT assay.

### 2.10. MAT Robustness for OMPC

To streamline lab transfers and facilitate QC processes, the RTU model of TLR Bioassay cells was used in this study to assess robustness of MAT. The critical method parameters, including cell seeding density, cell seeding time prior to the assay, assay incubation time, and NanoLuc detection time, were evaluated for assay robustness through a Design of Experiments (DOE) study (Table S1). In the DOE study, a four-factor D optimal custom design with random blocks was employed, treating the analysts and the days of the runs as random blocks. The study included 20 runs (plates) across five blocks, with each run testing OMPC samples at target pyrogenicity levels of 25%, 50%, 100%, and 150%, alongside reference standards.

### 2.11. Statistical Analysis

The percent relative pyrogenicity data were natural log (ln) transformed to meet normality assumption prior to all the analyses. The analyses were conducted using the R program (version 4.4.2) embedded with R studio (version 2024.12.0 Build 467).

Linearity assesses its ability (within a given range) to obtain test results which are directly proportional to the concentration (amount) of analyte in the sample. It was evaluated using linear regression analysis of the log transformed observed versus target relative pyrogenicity values for the simulated OMPC test samples.

Accuracy expresses the closeness of agreement between the value which is accepted either as a conventional true value or an accepted reference value and the value found. Since there was no pure analyte, accuracy was assessed with relative bias by comparing observed values vs. target value at each target level, defined as follows:

$$\%\;\mathrm{relative bias}=\left(\frac{\mathrm{Observed Value}}{\mathrm{Target Value}}-1\right)\times 100\%$$

Precision expresses the closeness of agreement (degree of scatter) between a series of measurements obtained from multiple samplings of the same homogeneous sample under the prescribed conditions. It was assessed by evaluating the variability of replicate measurements from the simulated samples at each target relative pyrogenicity level within a run and between-runs in the same laboratory. Within-a-run variability, also referred to repeatability, is the variability observed when measurements are taken in the same run. Between-runs variability arises from factors such as different analysts, different days, different reagent or assay media lots. The sum of the within-a-run and between-runs variability is the total variability, also termed as the intermediate precision (IP). Both repeatability and IP were typically expressed with percent relative standard deviation (%RSD) on the original scale of the % relative pyrogenicity,

$$\%\mathrm{RSD}=\sqrt{\exp({\mathrm{SD}}^{2})-1}\times 100,$$

where SD^2^ represents the within-a-run variance (repeatability) and the total variance (IP) estimates, respectively. The variance estimates can be obtained through variance component analysis (VCA) with restricted maximum likelihood estimation (REML) algorithm of log transformed relative pyrogenicity of the OMPC samples.

A reportable value is the final value obtained from an assay that reflects the relative pyrogenicity of a sample. This value is determined by calculating the geometric mean of replicate measurements of OMPC samples taken across multiple runs/plates of the assay. The variance of the reportable value, referred to as format variability, can be derived from variance estimates obtained through VCA. The final assay format is then selected based on the assessed format variability, ensuring that it meets the necessary reliability and consistency standards for reporting the relative pyrogenicity of a sample.

The assay range is the interval between the upper and lower levels of relative pyrogenicity in the samples for which it has been demonstrated that the analytical procedure has a suitable level of linearity, precision, and accuracy. It is derived based on the results from linearity, precision, and accuracy.

Robustness refers to a method’s ability to remain unaffected by small, deliberate variations in method parameters. The robustness of the MAT was evaluated based on the impact of varying method parameters on the relative pyrogenicity of the OMPC samples. A mixed effect analysis of variance (ANOVA) was employed for the evaluation. The analysis was conducted per target pyrogenicity levels. Assay robustness is concluded if the *p*-value for the significance test > 0.05 or the effect size of the factor is small compared to the overall mean. Robustness was also evaluated through the impact of varying method parameters on assay performance parameters: linearity, accuracy, and precision.

## 3. Results

### 3.1. Expression of TLR in Human Monocytic Cell Lines

**Figure 1:**
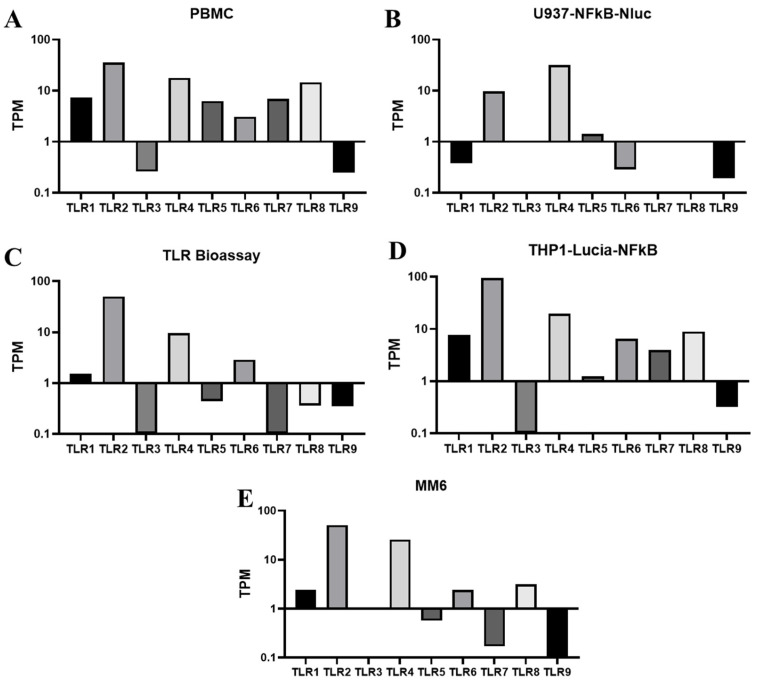
Normalized gene expressed levels (TPM) of TLR1-9 were determined by RNA seq for different types of human monocytic cells, including PBMCs (**A**), U937-NFκB-Nluc (**B**), TLR Bioassay (**C**), THP1-Lucia-NFκB (**D**), and MM6 (**E**) cells. TPM, transcripts per million.

Immune cells detect pyrogens, particularly microorganisms, which are the most likely pyrogenic contaminants in pharmaceutical products, through TLRs. The expression of TLR transcripts was assessed by RNA sequencing in various human monocytic cell lines, including U937-NFkB-NLuc, THP1-Lucia-NFkB, TLR Bioassay cells, and MM6. The expression levels of TLR1 through TLR9 were measured in these cells in the absence of stimulation to evaluate the transcription levels of the receptors for pathogen-associated molecular patterns (PAMPs) or pyrogens. PBMCs were used as a positive control, as these PAMP receptors, TLR1-9, have been shown to exhibit high expression in heterogeneous immune cells, (Figure 1A). Given the variability in the populations and yields of primary monocytes from PBMCs, THP-1, U937, and MM6 are frequently used human monocytic cell lines due to their similarities to primary monocytes and greater consistency in biological responses. Among these monocytic cell lines, THP1-Lucia-NFkB and TLR Bioassay cells, which are derived from THP-1 cells, exhibited a broad range of TLR expressions, encompassing all TLRs from TLR1 to TLR9, with particularly high levels of TLR2 and TLR4 (Figure 1C,D). U937-NFkB-NLuc expressed high levels of TLR4, moderate levels of TLR2, and low levels of TLR1 and TLR6 (Figure 1B). MM6 cells [19,20], the most commonly used monocytic cell line in MAT assays, demonstrated comparable TLR expression to the THP1 reporter cells but exhibited lower TLR3 expression (Figure 1E).

**Figure 2:**
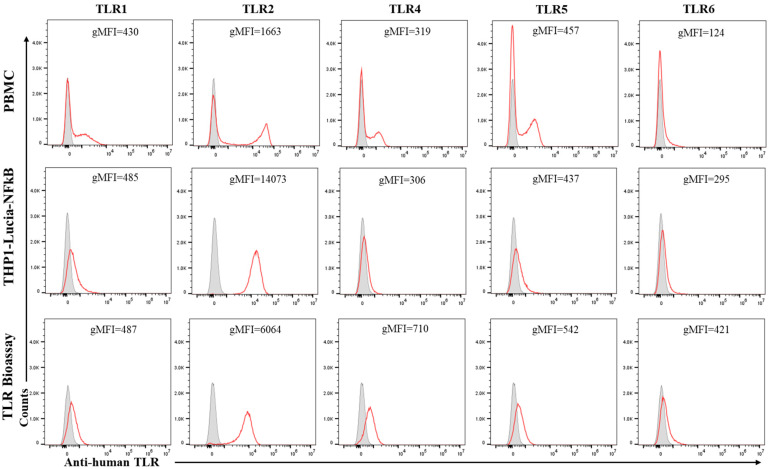
Expression of TLR1, TLR2, TLR4, TLR5, and TLR6 in THP-1-Lucia-NFκB cells, TLR Bioassay cells, and PBMCs. Representative flow cytometry histograms display signals from phycoerythrin (PE)-coupled human anti-TLR1, TLR2, TLR4, TLR5, and TLR6 (red unfilled) alongside their corresponding negative or isotype controls (gray filled). Geometric mean fluorescence intensity (gMFI) for each TLR was shown in the corresponding panel.

The protein expressions of the TLRs were further evaluated in the THP1 reporter cell lines (THP1-Lucia-NFkB and TLR Bioassay cells) using flow cytometry. As shown in Figure 2, similar to PBMCs, TLR1, TLR2, TLR4, TLR5, and TLR6 proteins were broadly expressed in TLR Bioassay cells, with predominant expression of TLR2. THP1-Lucia-NFkB cells exhibited comparable protein expression of these TLRs, although TLR4 protein expression was slightly lower compared to TLR Bioassay cells.

Overall, our data demonstrate that THP-1 reporter cell lines (THP1-Lucia-NFkB and TLR Bioassay cells) express most TLRs and have the potential to respond to their cognate ligands or pyrogens, such as lipopolysaccharide (LPS), lipoproteins, and flagellin.

### 3.2. Detection of Diverse Pyrogens by Reporter Cell Lines

**Figure 3:**
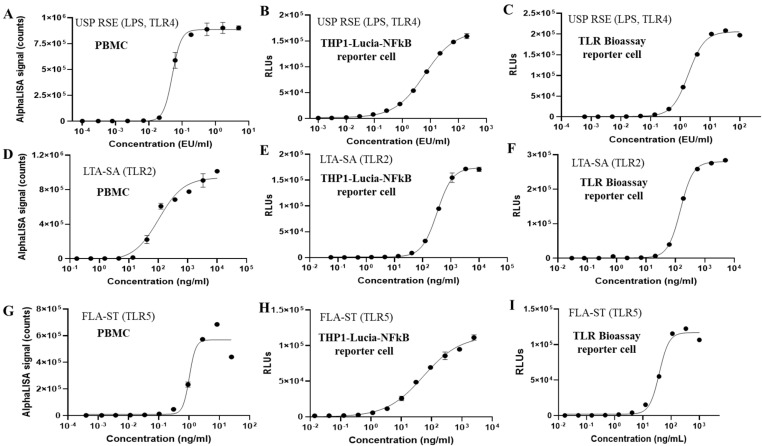
Dose response curves of PBMCs (**A**,**D**,**G**), THP1-Lucia-NFκB cells (**B**,**E**,**H**), and TLR Bioassay cells (**C**,**F**,**I**) following treatment with endotoxin and non-endotoxin pyrogens. The responses to the following stimuli were shown Endotoxin (USP RSE, TLR4) (**A**–**C**), LTA-SA (TLR2) (**D**–**F**), FLA-ST ultrapure (TLR5) (**G**–**I**). RTU was shown for TLR Bioassay cells and PBMCs. Cells from routine flask propagation were collected and used for THP1-Lucia-NFκB cells.

To evaluate the efficacy of human monocytic reporter cells as a testing system for monocyte activation tests (MAT), a range of endotoxin and non-endotoxin pyrogens were assessed, including the USP Reference Standard Endotoxin (USP RSE, TLR4), Pam3CSK4 (TLR1/2), HKLM (TLR2), LTA-SA (TLR2), FSL-1 (TLR2/6), Poly(I:C) (TLR3), FLA-ST ultrapure (TLR5), Imiquimod (TLR7), R848 (TLR7/8), and ODN2006 (TLR9). As illustrated in Figure 3B,C, both THP1-derived reporter cells, THP1-Lucia-NFkB and TLR Bioassay cells, exhibited strong reactivity to endotoxin (USP RSE). Additionally, for TLR Bioassay cells, both the propagation cell model (using cells from routine flask propagation) and the RTU model (using frozen vials directly) were tested, yielding similar responses in both models (Figure 3C,F,I and Figure S1). In contrast, the U937-derived reporter cell line (U937-NFkB-NLuc) demonstrated significantly lower sensitivity to endotoxin compared to the THP1 reporter cell lines (Figure S2A).

We also determined the limit of detection (LoD) for endotoxin across different test systems, defined as the minimum concentration that induced luminescence values exceeding three standard deviations above background levels. As shown in Figure 3 and Table 1, cryopreserved pooled PBMCs isolated from four donors, with over 10% monocytes, exhibited an LoD of approximately 0.01 EU/mL. And the LoD for endotoxin in THP1-Lucia-NFkB cells was approximately 0.02 EU/mL, while it was around 0.03 EU/mL in TLR Bioassay cells, highlighting their sensitivity as a cell source for endotoxin detection.

The endotoxin elicited a steep dose–response curve in PBMCs, whereas THP1-Lucia-NFkB and TLR Bioassay cells displayed a broader dynamic range. Given the difference in the sample-to-cell suspension volume ratio across different test systems, THP1-Lucia-NFkB cells could detect as low as ~0.04 EU/mL sample, TLR Bioassay cells could detect as low as 0.12 EU/mL per sample, and PBMCs detecting as low as ~0.02 EU/mL per sample. Consequently, the sensitivities of these reporter cell lines were either slightly lower or comparable to those of PBMCs, and notably higher than the sensitivity of the rabbit pyrogen test (RPT), which is approximately 5 EU/mL/kg or higher per sample [24].

In addition to endotoxin, both TLR reporter cell lines exhibited robust responses to non-endotoxin pyrogens (NEPs), including LTA-SA (TLR2), Pam3CSK4 (TLR1/2), HKLM (TLR2), FLS-1 (TLR2/6), and FLA-ST ultrapure (TLR5) (Figure 3 and Figures S1–S4). Importantly, TLR Bioassay cells demonstrated high sensitivity to TLR1, TLR2, and TLR6 ligands, comparable to that of PBMCs, and maintained stable, consistent performance through passage 25 (vendor guidance and we confirmed functionality up to passage 27 in our laboratory tests). However, both NF-κB reporter cell lines showed reduced sensitivity to TLR7/8 stimulation compared to PBMCs and exhibited limited responses to TLR3 and TLR9 ligands.

Based on our data both THP1-Lucia-NFkB and TLR Bioassay cells have the capacity to detect diverse endotoxin and NEP and are suitable to be used as test system for MAT assay. Due to simplicity of the procedure with single-addition reagent, extended luminescence output, and the choice of RTU cell model, the TLR Bioassay cells from Promega were selected as the test system in MAT assay development.

### 3.3. Development a MAT Method for Vaccines with Inherent Pyrogenicity

**Figure 4:**
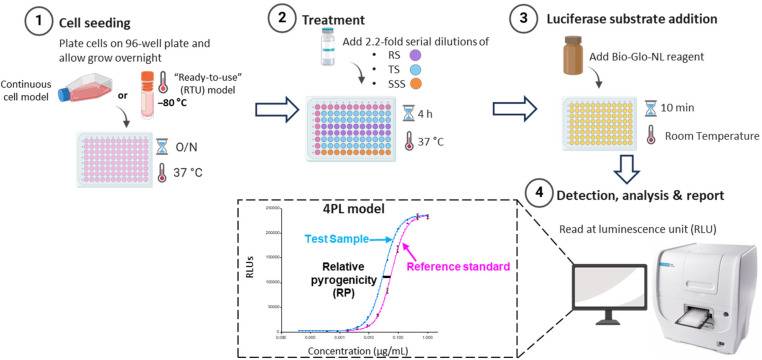
Schematic representation of reporter cell-based MAT. Steps 1–3 outline the procedure for the MAT assay, including the plate layout featuring the reference standard (RS), test sample (TS), and system suitability sample (SSS). Step 4 illustrates the data analysis conducted using a four-parameter logistic (4-PL) model, with relative pyrogenicity calculated through the PLA model in Gen5 software version 3.09. RLUs, relative luminescence units. Created with BioRender.com.

The outer membrane protein complex (OMPC) was produced and purified from the Gram-negative bacterium Neisseria meningitidis to enhance the immune response. The OMPC contains intrinsic endotoxin and non-endotoxin pyrogenic components, predominantly lipopolysaccharide (LPS) and lipoproteins, with a smaller proportion of flagellin. These components can activate TLR4, TLR2, and TLR5. As illustrated in Figure 4, in the MAT, TLR Bioassay cells were seeded in 96-well plates overnight before being treated with OMPC. After a 4 h treatment, luminescence was measured using a plate reader following the simple addition of luciferase reagent. With optimization of cell seeding density, sample treatment time, plate layout, and sample concentration (Table S3), an eleven-point, 2.2-fold dose–response curve was established for OMPC, and a 4-Parameter Logistic Model (4-PL) was fitted to the response data (RLUs) versus sample concentration. The relative pyrogenicity was assessed using parallel line analysis (PLA) in comparison to a reference lot after confirming the parallelism of the test sample and the reference standard.

**Figure 5:**
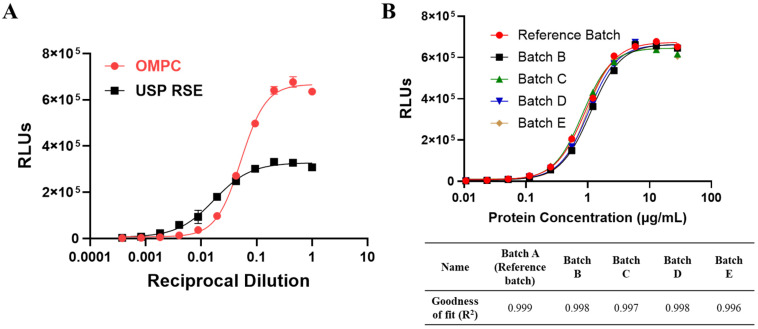
(**A**) Dose–response curves for TLR Bioassay cells treated with either OMPC or endotoxin (USP RSE). The maximum concentration of endotoxin tested is 100 EU/mL, while the highest concentration of OMPC protein is 56.7 µg/mL. (**B**) Dose–response curves and their fit to a four-parameter logistic (4-PL) model for screening and identifying five commercial batches against a reference batch.

In comparison to the endotoxin curves (USP RSE), the OMPC exhibited distinct dose–response behavior, characterized by a lack of parallelism (Figure 5A). According to Ph. Eur. 2.6.30 [12], Method 2 reference lot comparison test, which involves a comparison of the preparation to be examined with a validated reference lot of that preparation, is intended to be performed in cases where a preparation to be examined shows marked interference or because it contains or is believed to contain non-endotoxin contaminants. Consequently, the reference lot comparison test in Method 2 was identified as a more appropriate approach for OMPC. To identify a representative reference lot, five different commercial batches, all of which passed the Rabbit Pyrogen Test (RPT), were evaluated in the MAT assay, and the dose–response curves for these batches were fitted to the 4-PL model. As shown in Figure 5B, all five batches demonstrated a strong fit to the 4-PL model, with Batch A exhibiting a goodness of fit (R^2^) of 0.999, thereby identifying it as the reference batch. This batch also demonstrated parallelism with the other commercial batches.

### 3.4. Development of MAT Method for Vaccines with Inherent Pyrogenicity

**Figure 6:**
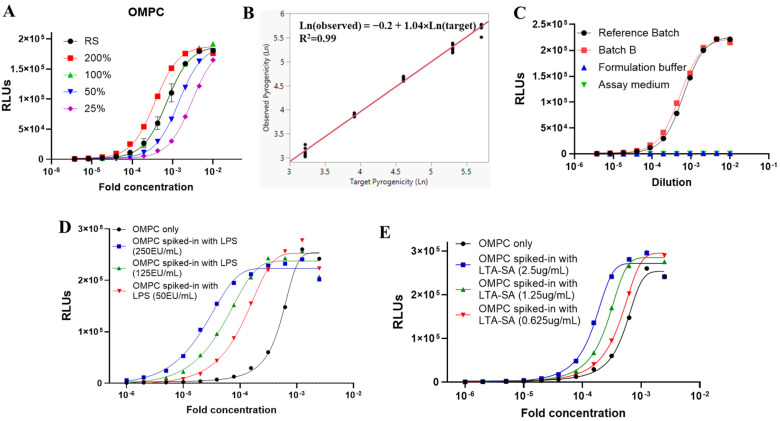
Performance characteristics of the MAT method for OMPC. (**A**) Dose–response curves illustrating the evaluation of relative pyrogenicity at 25%, 50%, 100%, and 200% compared to a reference batch. (**B**) Linear regression analysis depicting the logarithmic linear relationship between observed and expected relative pyrogenicity across five levels: 25%, 50%, 100%, 200%, and 300%. Each level is represented by 6 to 10 data points. (**C**) Dose–response curves for the formulation buffer in the MAT, both with and without OMPC. The formulation buffer did not interfere with the MAT method. (**D**) Dose–response curves for OMPC in the MAT, with varying concentrations of spiked pyrogens: endotoxin (LPS, USP RSE). (**E**) Dose–response curves for OMPC in the MAT with spiked LTA-SA at different concentrations. The concentrations indicated in the figures represent the final concentrations in the wells.

According to the guidelines set forth by the Ph. Eur. and the United States Food and Drug Administration (FDA), product-specific validation is required to determine whether a particular test substance or material is suitable for evaluation using the monocyte activation test (MAT) method. In alignment with the International Conference on Harmonization (ICH) guidelines for the Validation of Analytical Procedures (ICH Q2(R1)) [25], the reference lot comparison method in MAT should be validated as a quantitative content method. The following validation performance characteristics were assessed during method development: linearity, precision (including repeatability and intermediate precision), accuracy, range, and specificity. The identified commercial Batch A was utilized as the reference lot in the studies of MAT performance for OMPC. Test samples were prepared by diluting OMPC to various concentrations using formulation buffer, targeting five relative pyrogenicity levels (25%, 50%, 100%, 200%, and 300%) (Figure 6A,B). Figure 6B illustrated the derived linear function, “Ln(observed) = −0.20 + 1.04 * Ln(target),” with a coefficient of determination R^2^ of 0.99, indicating a strong linear relationship. The slope of 1.04 close to 1 suggested that the assay can almost achieve perfect proportional test results.

As shown in Table 2, the % relative bias for each level ranged from −0.4% to −10.3%, with an overall % relative bias of −2.0%. Precision, which reflects the closeness of agreement between a series of measurements obtained from multiple samplings of the same homogeneous sample, was evaluated through repeatability and intermediate precision across the range of 25% to 300% relative pyrogenicity. Repeatability represents within-day variability or plate-to-plate variability, while intermediate precision accounts for total assay variability, including plate-to-plate, day-to-day, and analyst-to-analyst variability. Precision was estimated using variance component analysis, with results shown in Table 2 indicating that the % relative standard deviation (RSD) for total variability was less than 10% at all five levels, and the overall repeatability % RSD and intermediate precision % RSD were both 6.9%. These results demonstrate that the MAT can reliably assess relative pyrogenicity ranging from 25% to 300% compared to the reference lot.

Specificity refers to the ability to unequivocally assess the test sample response in the presence of components that may be expected during sample testing, including impurities, degradants, and the sample matrix. The lack of interference from these matrix components must be demonstrated to establish specificity. The specificity of this assay was evaluated by analyzing formulation buffer with OMPC as the specific sample, while formulation buffer without OMPC served as the non-specific sample. As shown in Figure 6C, both the formulation buffer with OMPC (OMPC Batch B) and the formulation buffer without OMPC underwent the same dilution procedure to match the protein concentration of the OMPC reference lot. The formulation buffer without OMPC exhibited no dose response compared to the response elicited by the formulation buffer with OMPC and the reference lot. These data suggest that the MAT method is specific to OMPC and shows no interference from matrix components.

Furthermore, to investigate whether the MAT could detect potential pyrogen contaminants in the test samples, endotoxin (LPS) and non-endotoxin (LTA-SA) were spiked into the OMPC reference lot, and the corresponding responses were evaluated. As illustrated in Figure 6D,E, the dose–response curves of OMPC after spiking with different concentrations of LPS and LTA-SA exhibited shifts and distinct response behaviors compared to the OMPC reference lot. Notably, non-parallelism was observed after spiking with various endotoxin and non-endotoxin pyrogens, indicating that the MAT assay can not only monitor the consistency of the pyrogenic components in OMPC but also provide valuable information regarding potential pyrogen contamination during manufacturing.

### 3.5. MAT Robustness Study

**Figure 7:**
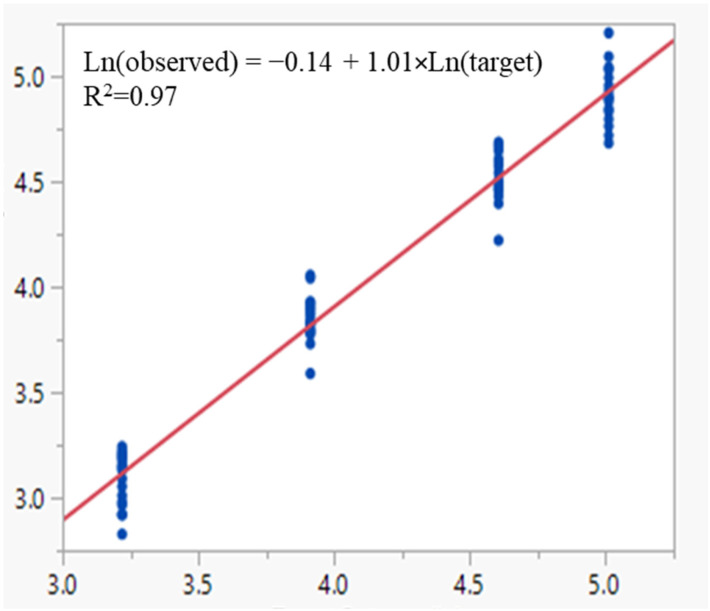
Linear regression of ln(observed) vs. ln(target) of relative pyrogenicity of the OMPC samples under different settings of the four robustness factors. The blue dots represent the observed relative pyrogenicity values of the OMPC samples. The red solid line represents the linear regression.

In accordance with ICH Q2(R1) [25], robustness assesses the method’s capacity to remain unaffected by small, but deliberate variations in method parameters, providing an indication of its reliability during routine use in the quality control (QC) laboratory. In the DOE study, the analysis of target levels at 25%, 50%, 100%, and 150% revealed that the only significant factor was the interaction between cell seeding density and cell seeding time at the 25% target level (*p* < 0.05) (Table S4). No significant terms were identified for the other target levels (Tables S5–S7). However, the small effect size of this interaction (estimate of 0.04) relative to the intercept (estimate if 3.06) suggests that its practical significance may be minimal. Furthermore, the assay performance was evaluated in terms of linearity, accuracy, and precision across various critical method parameters to confirm the assay robustness. As shown in Table 3, the % relative bias ranged from −6.2% to −11.7% across all four levels, with the corresponding 90% confidence intervals remaining within (−20%, 20%). The derived linear function, “ln(observed) = −0.14 + 1.01 × ln(target),” with an R^2^ of 0.97, indicates a strong linear relationship. The slope of 1.01 suggests that the assay can achieve proportional test results (Figure 7). The proportion bias is estimated as 1% ($=\left(2^{1.01-1}-1\right)\times 100$) per every 2-fold change in relative pyrogenicity. Precision was estimated using variance component analysis, with results shown in Table 4, which includes the variance estimates for repeatability and intermediate precision. Across all levels, the % RSD for intermediate precision was found to be 13.4%. These results indicate that the MAT performance was consistently not affected by all critical method parameters evaluated in this study, demonstrating that the MAT is robust for testing OMPC samples and is suitable for its intended purposes.

## 4. Discussion

Pyrogenicity is a critical safety characteristic that must be tested and controlled in all injectable pharmaceutical products, including vaccines. Historically, the RPT has been employed for the batch release of vaccines, particularly those with inherent pyrogenicity. However, due to inconsistencies and frequent false-positive results, coupled with the removal of RPT from the European Pharmacopoeia, there is an urgent need to develop a MAT to replace RPT for the batch release of vaccines [4,14]. One of the most challenging aspects of MAT is identifying an appropriate test system. Although PBMCs have been studied as the MAT test system for nearly two decades, their high variability among different donors and the complexity of the subsequent ELISA assays complicate the successful validation of the MAT method and its transfer to quality control (QC) laboratories [19].

Reporter cells have been widely utilized in cell-based assays to assess the mechanisms of action for biologic drugs, offering advantages such as high sensitivity, reduced variability, and a simplified workflow. While reporter cell lines have begun to be developed for MAT in recent years [26], very few have demonstrated their application for pyrogen release testing of vaccines with inherent pyrogenicity. Pyrogens, particularly pathogen-associated molecular patterns (PAMPs), can trigger fever by activating the human innate immune system. These patterns are recognized by pattern recognition receptors (PRRs), especially TLRs, leading to the release of inflammatory cytokines such as IL-6, IL-1, and TNF-α, which in turn induce fever. NF-κB serves as a central mediator for the induction of pro-inflammatory genes, including IL-1, IL-6, and TNF-α, in both innate and adaptive immune cells upon PAMP activation of TLRs. Therefore, NF-κB reporter cells derived from human monocytic cells present significant potential as a test system in MAT. Among various human monocytic cell lines, THP-1 cells, which naturally express most TLRs, have demonstrated high sensitivity in detecting diverse endotoxin and non-endotoxin pyrogens. In our study, the sensitivities of THP-1 reporter cells to TLR agonists were found to be slightly lower to those of PBMCs. In addition, the broader dynamic range of the THP-1 reporter cells could improve MAT performance, particularly for the Method 2 (lot comparison test) by keeping both test and reference responses within a measurable window, thereby reducing the need for repeated dilutions and increasing assay accuracy and precision [27]. It is important to note that the inherent variability of PBMCs from different donors poses challenges for consistent comparisons, therefore long-term supplies of PBMC lots are also of a significant concern. In addition to endotoxin, the reporter cell-based MAT has shown high sensitivity in detecting various non-endotoxin pyrogens, including Pam3CSK4 (a synthetic bacterial lipoprotein recognized by TLR1/2), LTA-SA (lipoteichoic acid, a major immune-stimulatory component of Gram-positive bacteria recognized by TLR2), flagellin (recognized by TLR5), FSL-1 (a synthetic lipoprotein derived from Gram-negative bacterium, recognized by TLR2/6), and R848 (an imidazoquinoline that mimics viral RNA and is recognized by TLR7/8). As the MAT is developed as an alternative to RPT, it is crucial that the MAT test system demonstrates equal or superior sensitivity in detecting both endotoxin and non-endotoxin pyrogens compared to RPT. Previous studies indicate that rabbits can detect approximately 5–10 EU/mL/kg of endotoxin and around 20 µg/mL/kg of LTA, while the reporter cell-based MAT can detect as low as approximately 0.12 EU/mL of endotoxin and 7.2 ng/mL of LTA-SA [20,28,29]. Reported minimum febrile doses for other TLR agonists in vivo are limited and highly variable, depending on agonist formulation, route of administration, and species. For example, R848 (a TLR7/8 agonist) elicited fever in mice after intraperitoneal injection at ~2–5 mg/kg [30,31], while flagellin-containing vaccines have produced febrile responses in humans at doses of roughly 2–10 µg following subcutaneous administration in some clinical trials [32,33]. Based on the existing data and reports for the minimum doses of TLR agonists inducing fever in vivo, our in vitro MAT demonstrated superior sensitivity for detecting both endotoxin and non-endotoxin pyrogens.

Under Ph. Eur. guidelines, product-specific validation of the MAT is required prior to its use for pyrogen testing before product release. It is essential to develop and validate the MAT method according to specific requirements to ensure its suitability, reliability, and robustness. Given that OMPC contains inherent pyrogenic components, interference cannot be diluted out within the maximum dilution volume, and the dose–response curve of OMPC does not parallel international endotoxin reference standards. Consequently, the reference lot comparison test (Method 2) has been determined to be the most appropriate method for OMPC. The observed non-parallelism with endotoxin has also been noted for other OMPC-based vaccines, likely due to the fact that endotoxin is not the only major pyrogenic component in OMPC, and different pyrogenic components activate various PRRs, such as TLR4, TLR2, and TLR5 [34]. In the reference lot comparison method, reference materials should be identified from the specific product and demonstrated to be safe and efficacious through clinical studies or representative data [12]. As the OMPC-based vaccine used in this study is a licensed product, the reference materials were screened and identified from several commercial batches that passed the RPT and are under quality control. The reference lot has also demonstrated a dose–response curve with a strong fit to the 4-Parameter Logistic (4PL) model (R^2^ ≥ 0.97) and parallelism with other representative batches. Any future reference lot should also be qualified and bridged under these criteria. The development of the reporter cell-based MAT for OMPC were conducted in accordance with the relevant requirements outlined in the ICH Q2(R1) guidelines [25] and Ph. Eur. 2.6.30; 2.6.40 [12,13]. The reporter cell-based MAT demonstrated excellent linearity (R^2^ ≥ 0.99), accuracy (relative bias % within ±10.3%), and precision (overall % RSD ≤ 6.9%). The combined data indicate that the assay can reliably report results within a range of 25–300%. The assay was shown to be specific for OMPC, with no responses observed in formulation buffer lacking OMPC material. Furthermore, when endotoxin or non-endotoxin pyrogenic contaminants were introduced into OMPC, non-parallelism in the dose–response curves was observed, indicating that the response to the test sample does not follow a consistent relationship with pyrogen contamination. This variability arises because pyrogen contaminants may interact with different receptors, pathways, or exhibit synergistic effects compared to the test sample, leading to variations in the overall immune response. Therefore, criteria for test samples should be established to ensure parallelism with the reference lot, and any samples failing to meet these criteria may indicate pyrogen contamination. Finally, the MAT assay demonstrated robustness, remaining unaffected by deliberate variations in cell densities, cell seeding time, assay incubation time, and NanoLuc detection time, thereby confirming its suitability for release testing. While the MAT described here was developed for OMPC, we will explore its adaptation using the reporter cell-based MAT for vaccines that include TLR-agonist adjuvants in accordance with regulatory guidance. If such vaccines have inherently high pyrogen content or produce interference that cannot be resolved within the maximum valid dilution (MVD), the Method 2 reference lot comparison method should be applied. A representative reference lot of the vaccine (including adjuvant), demonstrated to be safe and efficacious via appropriate preclinical and/or clinical studies, will be identified for comparative testing. Appropriate specifications will be established to determine product safety.

## 5. Conclusions

In summary, the human monocytic reporter cell line has proven to be an advantageous test system for the monocyte activation test (MAT), offering enhanced consistency, reduced variability, and greater simplicity compared to peripheral blood mononuclear cells (PBMCs) or other cell lines. The evaluation of performance characteristics demonstrates that the assay can reliably determine relative pyrogenicity across a broad range with high robustness and is ready for validation in a GxP laboratory. These advantages present significant benefits for future release testing in quality control (QC) laboratories and facilitate laboratory transfers. To our knowledge, this is the first report of a product-specific reference lot comparison test for a commercial vaccine with inherent pyrogenicity utilizing reporter cells in MAT. The design and development of the MAT method for OMPC-based vaccines, combined with its exceptional robustness, provide a valuable model for other pharmaceutical products exhibiting inherent pyrogenicity.

## Figures and Tables

**Table 1:** Summary of EC50 and sensitivity (limit of detection (LoD) or test sensitivity (TS)) for TLR agonists detected by PBMCs, THP1-Lucia-NFkB and TLR Bioassay cells. Notes: ^1^. The EC50 values were calculated using a four-parameter logistic (4PL) model. ^2^. The limit of detection (LoD) was defined as the lowest concentration that produced a signal greater than the blank (~8 negative controls) plus three standard deviations. ^3^. The test sensitivity (TS) is defined as the lowest concentration (actual data point on standard curve) of the pyrogen reference standard whose response exceeds the cut-off value (blank mean of ~8 negative controls plus three standard deviations). Values marked with “*” were derived from TS rather than LoD. The concentrations used in the wells were applied in the calculations described above.

TLR Agonist	Name	PBMCs		THP1-Lucia-NFkB		TLR Bioassay Cells	
		EC50 ^1^	LoD ^2^/TS ^3^	EC50 ^1^	LoD ^2^/TS ^3^	EC50 ^1^	LoD ^2^/TS ^3^
TLR4	USP-RSE (LPS) (EU/mL)	0.050	0.010	6.286	0.020	1.867	0.0323
TLR1/2	Pam3CSK4 (ng/mL)	3.689	0.063	27.83	0.457 *	2.945	0.0011 *
TLR2	HKLM (cells/mL)	2.84 × 10^6^	5.68 × 10^4^	1.50 × 10^8^	1.50 × 10^6^	5.94 × 10^7^	6.86 × 10^5^ *
TLR2	LTA-SA (ng/mL)	103.2	3.416	325.8	20.448	148.2	1.8
TLR2/6	FSL-1 (ng/mL)	0.1618	0.010	1.352	0.007	2.048	0.0027
TLR3	Poly(I:C) HMW (ng/mL)	4342	567.103	NC	NC	NC	NC
TLR3	Poly(I:C) LMW (ng/mL)	NC	16,666.67 *	NC	NC	NC	NC
TLR5	FLA-ST ultrapure (ng/mL)	1.328	0.085	53.42	0.117	37.810	1.372 *
TLR7	Imiquimod (ng/mL)	6460	321.404	NC	NC	NC	NC
TLR7/8	R848 (ng/mL)	206	4.670	3442	334.416	1.87 × 10^4^	1.50 × 10^3^
TLR9	ODN2006 (µM)	70.64	34.3 *	NC	NC	NC	NC

Abbreviations: EC50, half maximal effective concentration; LoD, limit of detection; TS, test sensitivity; NC, not calculable (EC50/LoD/TS could not be determined because a dose response curve was not obtained).

**Table 2:** Summary of precision and relative accuracy of the MAT method for OMPC. Precision was assessed as intermediate precision and repeatability at five levels (L1–L5, representing 25%, 50%, 100%, 200%, and 300%) and overall. Relative accuracy was expressed as relative bias at the same five levels and overall. Results were analyzed from 14 independent experiments. RSD, relative standard deviation.

Level	Intermediate Precision (%RSD)	Repeatability (%RSD)	Relative Bias (%)
L1 (25%)	8.5	8.1	−10.3
L2 (50%)	3.3	3.0	−1.2
L3 (100%)	3.3	3.3	4.6
L4 (200%)	7.7	7.7	−1.8
L5 (300%)	9.8	9.8	−0.4
Overall	6.9	6.9	−2.0

**Table 3:** Relative bias of the relative pyrogenicity of the OMPC samples at the four target levels. Geomean (%) is the geometric mean of observed relative pyrogenicity from 20 independent DOE runs, expressed as a percentage of the nominal target. 90% Lower CL (%) and 90% Upper CL (%) are the lower and upper bounds, respectively, of the 90% confidence interval for the percent relative bias.

Target (%)	N	Geomean (%)	% Relative Bias	90% Lower CL of Relative Bias (%)	90% Upper CL of Relative Bias (%)
25	20	22.1	−11.7	−15.6	−7.5
50	20	46.9	−6.2	−9.9	−2.2
100	20	91.6	−8.4	−12.3	−4.3
150	20	136.3	−9.2	−13.5	−4.6

**Table 4:** Variance Component Analysis of Precision (Repeatability and Intermediate Precision) under different settings of the four robustness factors. “Day[Analyst]” indicates day-to-day variability attributed to the analyst, while “Residual” represents repeatability, reflecting within-day variability or plate-to-plate variability. $\%RSD=\sqrt{\exp\left(Variance\right)-1}\times 100$, where variance represents the variance estimates for the corresponding components. Results were analyzed from 20 runs in a Design of Experiments (DOE) study. RSD, relative standard deviation.

Target (%)	% RSD			
	Analyst	Day[Analyst]	Residual	Total
25	14.5	0	5.9	15.7
50	8.2	2.8	8.5	12.1
100	10.8	0	8.2	13.6
150	1.8	0	12.5	12.7
Combined	9.6	0.8	9.3	13.4

## Data Availability

The data supporting this study are available from the corresponding author upon reasonable request.

## Supplementary Materials

The following supporting information can be downloaded at: https://www.mdpi.com/article/10.3390/vaccines13101009/s1. Table S1. Method parameters evaluated in the MAT robustness DoE study for OMPC. Figure S1. Dose response curves of TLR Bioassay cells using continuous cell model (using cells from routine flask propagation) following treatment with endotoxin and non-endotoxin pyrogens. The responses to the following stimuli were shown Endotoxin (USP RSE, TLR4) (A), Pam3CSK4 (TLR1/2) (B), HKLM (TLR2) (C), LTA-SA (TLR2) (D), FSL-1 (TLR2/6) (E), FLA-ST ultrapure (TLR5) (F), and R848 (TLR7/8) (G). Figure S2. Dose response curves of U937-NFkB-NLuc cells using continuous cell model (using cells from routine flask propagation) following treatment with endotoxin and non-endotoxin pyrogens. The responses to the following stimuli were shown Endotoxin (USP RSE, TLR4) (A), Pam3CSK4 (TLR1/2) (B), LTA-SA (TLR2) (C), FSL-1 (TLR2/6) (D), and FLA-ST ultrapure (TLR5) (E). The concentrations in wells were used to generate the dose-response curves for each TLR agonist. Figure S3. Dose response curves of PBMCs treated with diverse non-endotoxin pyrogens are shown for Pam3CSK4 (TLR1/2) (A), HKLM (TLR2) (B), FSL-1 (TLR2/6) (C), Poly(I:C) HMW (TLR3) (D), R848 (TLR7/8) (E) and ODN 2006(TLR9) (F). The concentrations in wells were used to generate the dose-response curves for each TLR agonist. Figure S4. Dose response curves of THP1-Lucia-NFκB cells using continuous cell model (using cells from routine flask propagation) following treatment with diverse non-endotoxin pyrogens. The responses to the following stimuli were shown Pam3CSK4 (TLR1/2) (A), HKLM (TLR2) (B), FSL-1 (TLR2/6) (C), and R848 (TLR7/8) (D). The concentrations in wells were used to generate the dose-response curves for each TLR agonist. Table S2. Summary of EC50 and sensitivity (limit of detection (LoD) or test sensitivity (TS)) for TLR agonists detected by U937-NFkB-NLuc cells. Notes: 1. The EC50 values were calculated using a four-parameter logistic (4PL) model. 2. The limit of detection (LoD) was defined as the lowest concentration that produced a signal greater than the blank (~8 negative controls) plus three standard deviations. 3. The test sensitivity (TS) is defined as the lowest concentration (actual data point on standard curve) of the pyrogen reference standard whose response exceeds the cut-off value (blank mean of ~8 negative controls plus three standard deviations). Values marked with “*” were derived from TS rather than LoD. The concentrations used in the wells were applied in the calculations described above. Table S3. Method parameters optimization and robustness for MAT for OMPC. Table S4: The effect of parameters at target level of 25% relative pyrogenicity. Table S5: The effect of parameters at target level of 50% relative pyrogenicity. Table S6: The effect of parameters at target level of 100% relative pyrogenicity. Table S7: The effect of parameters at target level of 150% relative pyrogenicity.
