# Supplementary material for: Advancing Pyrogen Testing for Vaccines with Inherent Pyrogenicity: Development of a Novel Reporter Cell-Based Monocyte Activation Test (MAT)
Source: Vaccines (Basel). 2025 Sep 26;13(10):1009. doi: 10.3390/vaccines13101009 (PMC12568226; doi:10.3390/vaccines13101009)
Supplement: Supplementary file 1 [file vaccines-13-01009-s001.zip › vaccines-3860123-supplementary.pdf]

Proprietary

Advancing Pyrogen Testing for Vaccines with Inherent Pyrogenicity: Development of a Novel Reporter Cell-Based Monocyte Activation Test (MAT)

Sijia Yi[1*], Jenny Xu[1], Liping Song[2], Frank Celeste[1], Christopher J Wang[1], Melissa C Whiteman[1]

[1] **Analytical Research and Development, Merck & Co., Inc., West Point, PA 19486, USA**

[2] **Biostatistics and Research Decision Sciences, Merck & Co., Inc., West Point, PA 19486, USA**

* Author to whom correspondence should be addressed

Supplementary information

Factor	Levels
Cell Seeding Density (cells/well)	2 x10^4, 2.5 x10^4, and 3.5 x10^4
Cell Seeding Time (hours)	18 and 24
Assay Incubation Time (hours)	3.5, 4, and 5
NanoLuc Detection Time (minutes)	5and 15

Table S1. Method parameters evaluated in the MAT robustness DoE study for OMPC

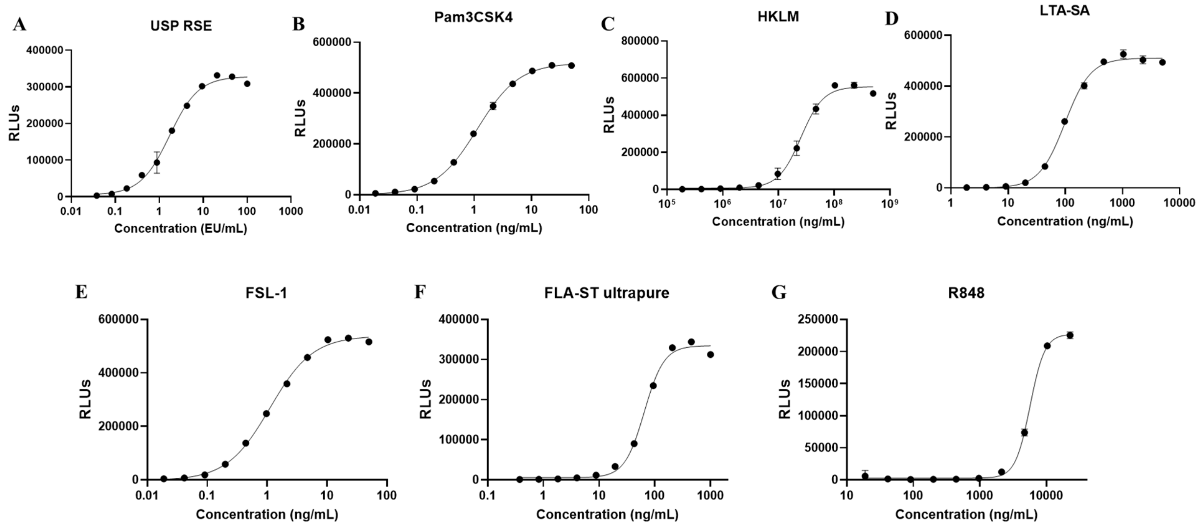

Figure S1. Dose response curves of TLR Bioassay cells using continuous cell model (using cells from routine flask propagation) following treatment with endotoxin and non-endotoxin pyrogens. The responses to the following stimuli were shown Endotoxin (USP RSE, TLR4) (A), Pam3CSK4 (TLR1/2) (B), HKLM (TLR2) (C), LTA-SA (TLR2) (D), FSL-1 (TLR2/6) (E), FLA-ST ultrapure (TLR5) (F), and R848 (TLR7/8) (G).

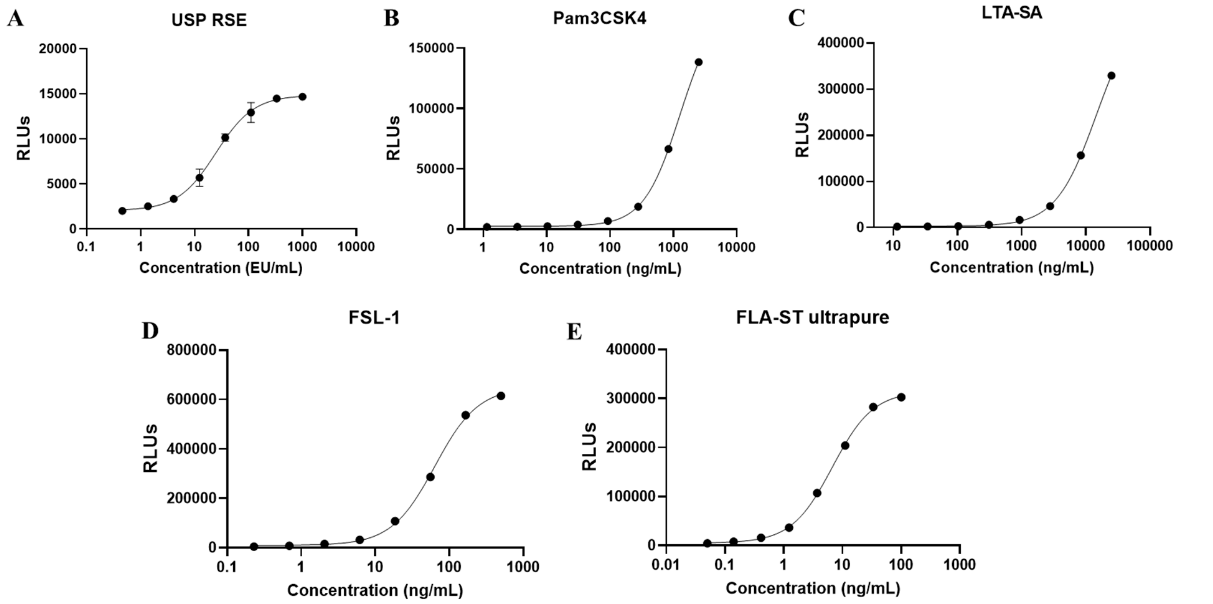

Figure S2. Dose response curves of U937-NFkB-NLuc cells using continuous cell model (using cells from routine flask propagation) following treatment with endotoxin and non-endotoxin pyrogens. The responses to the following stimuli were shown Endotoxin (USP RSE, TLR4) (A), Pam3CSK4 (TLR1/2) (B), LTA-SA (TLR2) (C), FSL-1 (TLR2/6) (D), and FLA-ST ultrapure (TLR5) (E). The concentrations in wells were used to generate the dose-response curves for each TLR agonist.

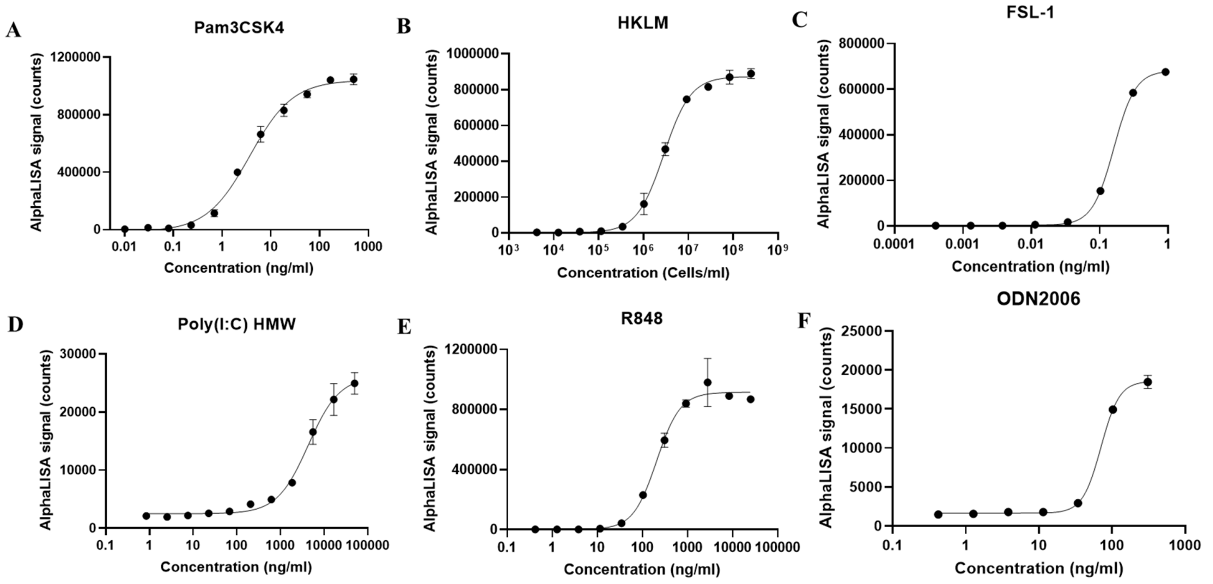

Figure S3. Dose response curves of PBMCs treated with diverse non-endotoxin pyrogens are shown for Pam3CSK4 (TLR1/2) (A), HKLM (TLR2) (B), FSL-1 (TLR2/6) (C), Poly(I:C) HMW (TLR3) (D), R848 (TLR7/8) (E) and ODN 2006(TLR9) (F). The concentrations in wells were used to generate the dose-response curves for each TLR agonist.

Proprietary

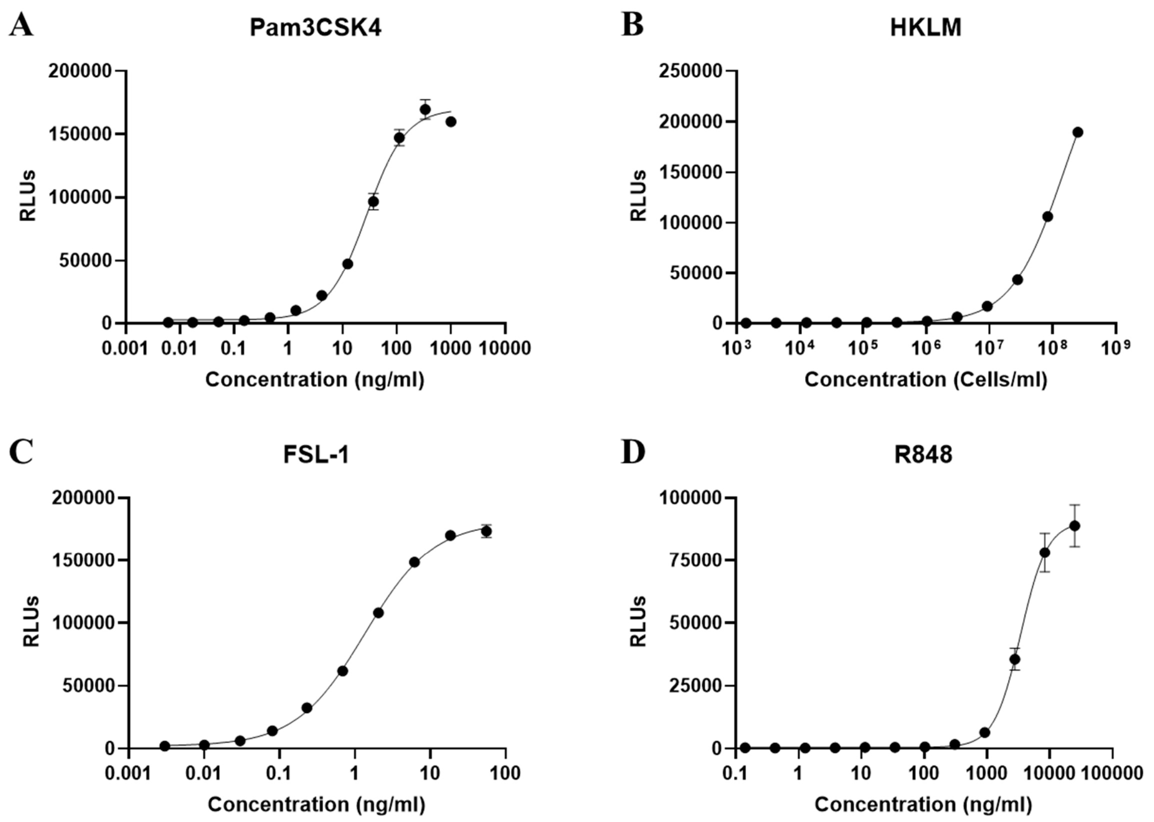

Figure S4. Dose response curves of THP1-Lucia-NFκB cells using continuous cell model (using cells from routine flask propagation) following treatment with diverse non-endotoxin pyrogens. The responses to the following stimuli were shown Pam3CSK4 (TLR1/2) (A), HKLM (TLR2) (B), FSL-1 (TLR2/6) (C), and R848 (TLR7/8) (D). The concentrations in wells were used to generate the dose-response curves for each TLR agonist.

TLR agonist	Name	U937-NFkB-NLuc	
		EC50[1]	LoD[2]/TS[3]
**TLR4**	USP-RSE (LPS) (EU/ml)	24.90	0.85
**TLR1/2**	Pam3CSK4 (ng/ml)	1263	10.29*
TLR2	HKLM (cells/ml)	1.74E+09	4.80E+05
**TLR2**	LTA-SA (ng/ml)	14600	34.30*
**TLR2/6**	FSL-1 (ng/ml)	63.7	0.23
TLR3	Poly(I:C) HMW (ng/ml)	NC	NC
TLR3	Poly(I:C) LMW (ng/ml)	NC	NC

**TLR5**	FLA-ST ultrapure (ng/ml)	6.73	0.05
TLR7	Imiquimod (ng/ml)	NC	NC
TLR7/8	ssRNA40 (ng/ml)	NC	NC
TLR9	ODN2006 (μM)	2772.5	377.16

Table S2. Summary of EC50 and sensitivity (limit of detection (LoD) or test sensitivity (TS)) for TLR agonists detected by U937-NFkB-NLuc cells. Notes: 1. The EC50 values were calculated using a four-parameter logistic (4PL) model. 2. The limit of detection (LoD) was defined as the lowest concentration that produced a signal greater than the blank (~8 negative controls) plus three standard deviations. 3. The test sensitivity (TS) is defined as the lowest concentration (actual data point on standard curve) of the pyrogen reference standard whose response exceeds the cut-off value (blank mean of ~8 negative controls plus three standard deviations). Values marked with "*" were derived from TS rather than LoD. The concentrations used in the wells were applied in the calculations described above.

Abbreviation: EC50, half maximal effective concentration; LoD, limit of detection; TS, test sensitivity; NC, not calculable (EC50/LoD/TS could not be determined because a dose response curve was not obtained).

Parameter	Optimal	Tested in robustness study
Cell Seeding Density (cells/well)	3 $\times10^4$	2 $\times10^4$ to 3.5 $\times10^4$
Cell Seeding Time (hours)	22	18-24
Assay Incubation Time (hours)	4	3.5-5
NanoLuc Detection Time (minutes)	10	5-15
Edge effects	Avoid row A and column 1	

Table S3. Method parameters optimization and robustness for MAT for OMPC.

Term	Estimate	Std Error	DFDen	t Ratio	Prob>|t|
Intercept	3.0597076	0.056321	5.765	54.33	<.0001*
Cell Seeding Density	-0.017268	0.010064	3.073	-1.72	0.1825
Cell Seeding Density*Cell Seeding Density	-0.060458	0.039143	3.102	-1.54	0.2172
Cell Seeding Time	-0.015286	0.010171	3.116	-1.50	0.2266
Assay Incubation Time	-0.023035	0.010064	3.073	-2.29	0.1040
Assay Incubation Time*Assay Incubation Time	0.0994702	0.039143	3.102	2.54	0.0818
NanoLuc Detection Time	0.0056413	0.010135	3.099	0.56	0.6155
Cell Seeding Density*Cell Seeding Time	0.0364431	0.011239	3.137	3.24	0.0448*
Cell Seeding Density*Assay Incubation Time	0.0132784	0.010504	3.088	1.26	0.2932
Cell Seeding Density*NanoLuc Detection Time	0.0171143	0.010124	3.076	1.69	0.1873

Term	Estimate	Std Error	DFDen	t Ratio	Prob>|t|
Cell Seeding Time*Assay Incubation Time	-0.013052	0.011239	3.137	-1.16	0.3262
Cell Seeding Time*NanoLuc Detection Time	-0.017365	0.010276	3.111	-1.69	0.1864
Assay Incubation Time*NanoLuc Detection Time	-0.01138	0.010124	3.076	-1.12	0.3410

Table S4: The effect of parameters at target level of 25% relative pyrogenicity.

Term	Estimate	Std Error	DFDen	t Ratio	Prob>|t|
Intercept	3.8558533	0.092456	5.575	41.70	<.0001*
Cell Seeding Density	-0.011583	0.032849	3.747	-0.35	0.7433
Cell Seeding Density*Cell Seeding Density	-0.00678	0.127305	4.11	-0.05	0.9600
Cell Seeding Time	-0.018842	0.033918	4.596	-0.56	0.6045
Assay Incubation Time	0.0092371	0.032849	3.747	0.28	0.7934
Assay Incubation Time*Assay Incubation Time	-0.003137	0.127305	4.11	-0.02	0.9815
NanoLuc Detection Time	-0.003565	0.033561	4.204	-0.11	0.9203
Cell Seeding Density*Cell Seeding Time	-0.009128	0.036663	4.72	-0.25	0.8139
Cell Seeding Density*Assay Incubation Time	-0.012001	0.034791	4.063	-0.34	0.7473
Cell Seeding Density*NanoLuc Detection Time	0.0146527	0.033077	3.798	0.44	0.6818
Cell Seeding Time*Assay Incubation Time	-0.024959	0.036663	4.72	-0.68	0.5280
Cell Seeding Time*NanoLuc Detection Time	-0.040316	0.033556	4.285	-1.20	0.2917
Assay Incubation Time*NanoLuc Detection Time	0.0005848	0.033077	3.798	0.02	0.9868

Table S5: The effect of parameters at target level of 50% relative pyrogenicity

Term	Estimate	Std Error	DFDen	t Ratio	Prob>|t|
Intercept	4.5309384	0.073726	5.792	61.46	<.0001*
Cell Seeding Density	-0.01617	0.025673	3.809	-0.63	0.5646
Cell Seeding Density*Cell Seeding Density	0.0015041	0.099691	4.118	0.02	0.9887
Cell Seeding Time	-0.008139	0.026349	4.465	-0.31	0.7713
Assay Incubation Time	0.0053879	0.025673	3.809	0.21	0.8445
Assay Incubation Time*Assay Incubation Time	-0.021624	0.099691	4.118	-0.22	0.8386
NanoLuc Detection Time	-0.040492	0.026097	4.165	-1.55	0.1929
Cell Seeding Density*Cell Seeding Time	0.0430246	0.028693	4.602	1.50	0.1990
Cell Seeding Density*Assay Incubation Time	0.0127879	0.027062	4.051	0.47	0.6609
Cell Seeding Density*NanoLuc Detection Time	0.0161336	0.025853	3.851	0.62	0.5676
Cell Seeding Time*Assay Incubation Time	-0.010982	0.028693	4.602	-0.38	0.7190
Cell Seeding Time*NanoLuc Detection Time	-0.032814	0.026238	4.254	-1.25	0.2755
Assay Incubation Time*NanoLuc Detection Time	-0.016335	0.025853	3.851	-0.63	0.5630

Table S6: The effect of parameters at target level of 100% relative pyrogenicity

Term	Estimate	Std Error	DFDen	t Ratio	Prob>|t|
Intercept	4.8722159	0.092364	7	52.75	<.0001*
Cell Seeding Density	-0.03219	0.035001	7	-0.92	0.3883
Cell Seeding Density*Cell Seeding Density	-0.033591	0.130024	7	-0.26	0.8036
Cell Seeding Time	-0.022355	0.032356	7	-0.69	0.5119
Assay Incubation Time	-0.014882	0.035001	7	-0.43	0.6835
Assay Incubation Time*Assay Incubation Time	0.0813851	0.130024	7	0.63	0.5512
NanoLuc Detection Time	0.0108603	0.033506	7	0.32	0.7553
Cell Seeding Density*Cell Seeding Time	-0.009666	0.035001	7	-0.28	0.7904
Cell Seeding Density*Assay Incubation Time	-0.018384	0.035318	7	-0.52	0.6187
Cell Seeding Density*NanoLuc Detection Time	0.0291405	0.035001	7	0.83	0.4326
Cell Seeding Time*Assay Incubation Time	-0.055483	0.035001	7	-1.59	0.1569
Cell Seeding Time*NanoLuc Detection Time	-0.053945	0.033506	7	-1.61	0.1514
Assay Incubation Time*NanoLuc Detection Time	0.0003229	0.035001	7	0.01	0.9929

Table S7: The effect of parameters at target level of 150% relative pyrogenicity.

Notes: The results of Table S4-S7 were analyzed from 20 runs in a Design of Experiments (DOE) study. Term refers to the parameter to be evaluated. Std Error refers to the standard error of the parameter estimate. DFDen refers to the degrees of freedom of the denominator, the residual (error) degrees of the freedom of the model. T Ratio is the t statistic (the ratio of the estimate of the parameter and its standard error. Prob>|t| represents the probably of observing the t Ratio under the null hypothesis that the true effect is zero.
